# Germline *ETV6* Mutations Confer Susceptibility to Acute Lymphoblastic Leukemia and Thrombocytopenia

**DOI:** 10.1371/journal.pgen.1005262

**Published:** 2015-06-23

**Authors:** Sabine Topka, Joseph Vijai, Michael F. Walsh, Lauren Jacobs, Ann Maria, Danylo Villano, Pragna Gaddam, Gang Wu, Rose B. McGee, Emily Quinn, Hiroto Inaba, Christine Hartford, Ching-hon Pui, Alberto Pappo, Michael Edmonson, Michael Y. Zhang, Polina Stepensky, Peter Steinherz, Kasmintan Schrader, Anne Lincoln, James Bussel, Steve M. Lipkin, Yehuda Goldgur, Mira Harit, Zsofia K. Stadler, Charles Mullighan, Michael Weintraub, Akiko Shimamura, Jinghui Zhang, James R. Downing, Kim E. Nichols, Kenneth Offit

**Affiliations:** 1 Department of Medicine, Memorial Sloan Kettering Cancer Center (MSKCC), New York, New York, United States of America; 2 Cancer Biology and Genetics Program, Sloan Kettering Institute, New York, New York, United States of America; 3 St Jude Children’s Research Hospital (SJCRH), Memphis, Tennessee, United States of America; 4 Fred Hutchinson Cancer Research Center and University of Washington, Seattle, Washington, United States of America; 5 Pediatric Hematology/Oncology Department, Hadassah-Hebrew University Medical Center, Jerusalem, Israel; 6 University of British Columbia, Vancouver, British Columbia, Canada; 7 Weill Cornell Medical College, New York, New York, United States of America; 8 Structural Biology Program, Sloan Kettering Institute, New York, New York, United States of America; 9 Seattle Children’s Hospital, Seattle, Washington, United States of America

## Abstract

Somatic mutations affecting *ETV6* often occur in acute lymphoblastic leukemia (ALL), the most common childhood malignancy. The genetic factors that predispose to ALL remain poorly understood. Here we identify a novel germline *ETV6* p. L349P mutation in a kindred affected by thrombocytopenia and ALL. A second *ETV6* p. N385fs mutation was identified in an unrelated kindred characterized by thrombocytopenia, ALL and secondary myelodysplasia/acute myeloid leukemia. Leukemic cells from the proband in the second kindred showed deletion of wild type *ETV6* with retention of the *ETV6* p. N385fs. Enforced expression of the *ETV6* mutants revealed normal transcript and protein levels, but impaired nuclear localization. Accordingly, these mutants exhibited significantly reduced ability to regulate the transcription of *ETV6* target genes. Our findings highlight a novel role for *ETV6* in leukemia predisposition.

## Author Summary

Inherited mutations of transcription factors have recently been associated with susceptibility to acute leukemia. Here we report two unrelated kindreds with inherited mutations in *ETV6*, the gene encoding the transcription factor ETS variant 6. These families were characterized by a low platelet count (thrombocytopenia) and acute lymphoblastic leukemia (ALL). Sequencing a panel of genes identified germline *ETV6* mutations associated with leukemia and thrombocytopenia in multiple individuals tested. In one family, there was a substitution within the DNA binding domain of *ETV6*, termed L349P, and in the second there were five base pairs missing in *ETV6* (N385fs), causing an abnormally truncated protein. We overexpressed the *ETV6* mutants in the HeLa cell line and measured protein levels and localization within the cells. Instead of localizing to the nucleus, as expected for a transcription factor, the mutant proteins were found in the cytoplasm. The mutant proteins also showed decreased ability to regulate the expression of other genes typically suppressed by *ETV6*. These findings suggest that germline *ETV6* mutations cause a new type of heritable leukemia. This discovery makes possible the pre-symptomatic diagnosis of leukemia susceptibility in families with germline *ETV6* mutations, and also provides new information on the causes of leukemia.

## Introduction

Acute leukemias comprise the most common form of pediatric cancer, among which acute lymphoblastic leukemia (ALL) makes up 80–85% of the cases[1,2]. It is well recognized that a proportion of affected children develop the disease due to an underlying predisposition. The currently recognized genes responsible for autosomal dominant transmission of childhood leukemia include *TP53*, *CEBPA*, *PAX5* and *GATA-2*[3–8]. Occasionally, acute leukemia presents in the context of thrombocytopenia. Consistent with this feature, several heritable thrombocytopenia syndromes are known to exist, some of which are associated with an increased incidence of leukemia. Genes associated with these syndromes include *RUNX1*, *ANKRD26*, *GATA1*, *MPL*, *HOXA11* and *RMB8A*[9–16]. Despite the identification of these genes, there remain many cases for which the underlying mechanism remains unexplained. In this study, we analyzed one large kindred and one parent-child trio, both affected by ALL and thrombocytopenia. By exome sequencing and also sequencing plausible candidate genes such as those involved in B-lymphocyte development and differentiation, we identified germline mutations in the transcription factor *ETV6* that co-segregated with disease in each kindred. Functional studies support a pathogenic role for the observed mutations, both of which affect the DNA binding domain. These findings are consistent with independent observations describing additional kindreds characterized by thrombocytopenia and predisposition to hematopoietic malignancy[17,18] and provide insights into the mechanisms of leukemia susceptibility and clinical phenotypes associated with germline *ETV6* mutations[19].

## Results/Discussion

### Phenotypic features of the kindreds

**Fig 1:**
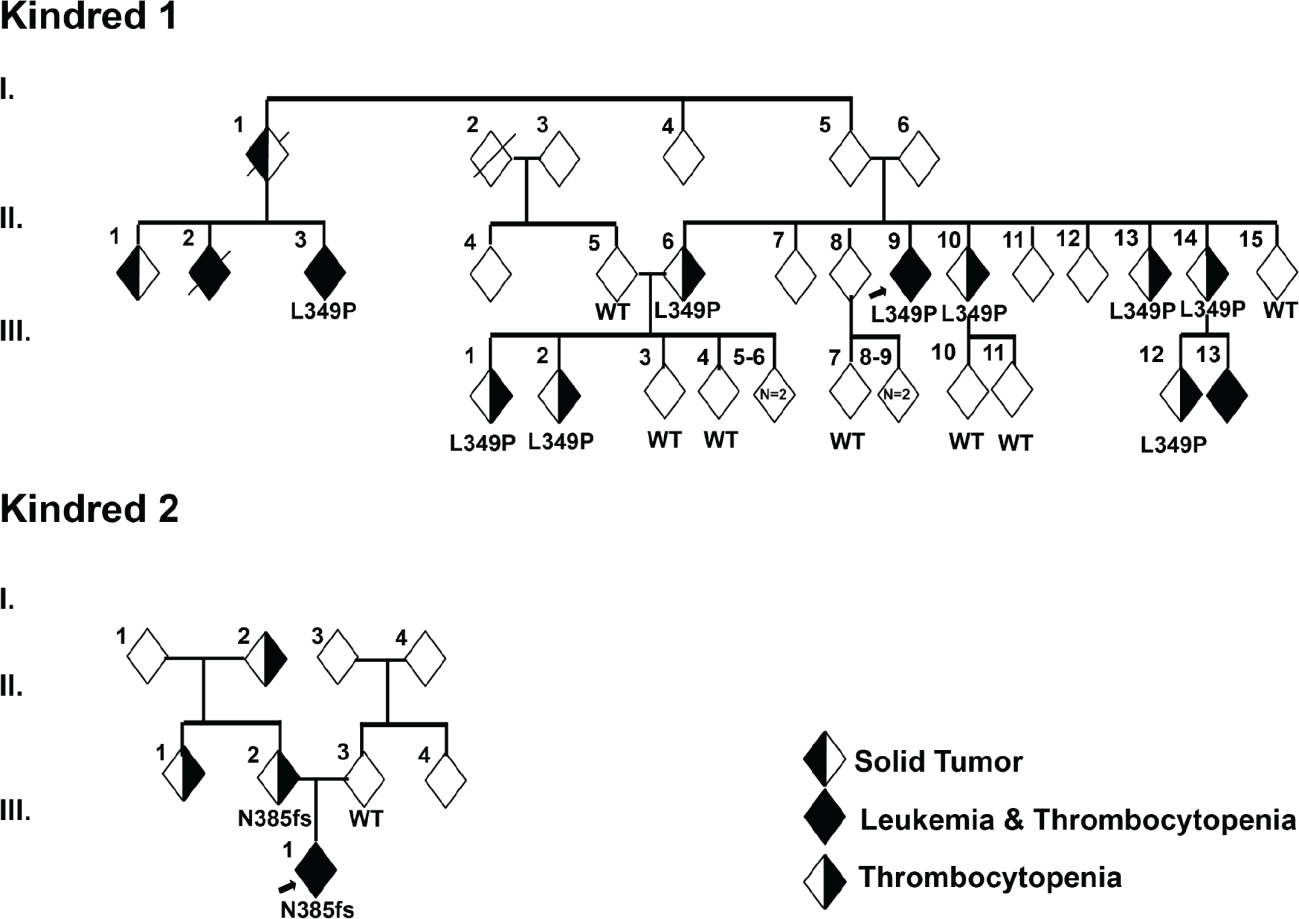
Identification of germline mutations in *ETV6* in 2 unrelated kindreds. (a) In Kindred 1, targeted sequencing identified a germline *ETV6* L349P mutation. Sequencing was performed on 9 individuals including the proband (arrow) affected with thrombocytopenia and/or ALL and 7 unaffected individuals as noted in Table 1. (b) In Kindred 2, clinical whole exome sequencing was performed on the proband (arrow) with ALL, MDS and AML, the mother with thrombocytopenia as well as the unaffected father. An *ETV6* N385fs mutation was identified. In both kindreds, the *ETV6* mutations segregated with disease.

As part of a collaborative study focusing on pedigree analysis and gene discovery in childhood leukemia, we identified a Polish/Moroccan kindred in which 10 individuals developed thrombocytopenia and 4 individuals developed thrombocytopenia and ALL (Kindred 1 in Fig 1A). In the 3 ALL cases in Kindred 1 for whom flow-cytometric data were available, all were of the pre-B-ALL subtype. In 3 cases with thrombocytopenia and no evidence of ALL, the mean corpuscular volume (MCV) was decreased in 1 case and normal in 2 others (Table 1). In 2 individuals with no evidence of hematologic abnormalities, there was a history of renal cell cancer and duodenal adenocarcinoma. A second unrelated Western European/Native American family was identified in which a child developed ALL followed by myelodysplastic syndrome and acute myeloid leukemia (AML). This child’s mother, maternal aunt and maternal grandfather exhibited thrombocytopenia (Kindred 2 in Fig 1B). This patient was evaluated by a geneticist due to subtle dysmorphic features; however, clinical assessment did not suggest a known genetic syndrome, and microarray and karyotype did not reveal any large deletions, rearrangements or other structural chromosomal abnormalities.

**Table 1:** Clinical features of individuals in the study kindreds.

Kindred	ETV6 alteration status	Individual	Cytopenias	Platelet count (per L)	MCV (fL)	Flow cytometry	Malignancies	Additional features
1	Not sequenced	I.1	Unknown	not available		n/a	RCC	
1	Not sequenced	II.1	Unknown	not available		n/a	Duodenal Adenocarcinoma	
1	Not sequenced	II.2	Thrombocytopenia	11 x 10^9^		n/a	ALL	
1	L349P	II.3	Thrombocytopenia	not available		CD19+,CD20+,CD22+,CD34+,TdT+	ALL	
1	L349P	II.6	Thrombocytopenia, anemia	8 x 10^9^		n/a		Arthritis
1	L349P	II.9	Thrombocytopenia	not available		CD10+,CD20+,CD22+,TdT+	ALL	
1	L349P	II.10	Thrombocytopenia	53 x 10^9^	85	n/a		Ankylosing spondylitis, uveitis
1	L349P	II.13	Thrombocytopenia	not available		n/a		
1	L349P	II.14	Thrombocytopenia	not available		n/a		
1	L349P	III.1	Thrombocytopenia	75 x 10^9^	85.8	n/a		Secondary amenorrhea
1	L349P	III.2	Thrombocytopenia, anemia	13 x 10^9^	79	n/a		
1	L349P	III.12	Thrombocytopenia	not available		n/a		Cleft lip/palate
1	Not sequenced	III.13	Thrombocytopenia	not available		CD10+,CD20+,CD79a+,TdT+	Pancytopenia, MDS, ALL	Cleft lip/palate
2	Not sequenced	I.2	Thrombocytopenia	not available		n/a		
2	Not sequenced	II.1	Thrombocytopenia	not available		n/a		
2	N385fs	II.2	Thrombocytopenia	not available		n/a		
2	N385fs	III.1	Thrombocytopenia	not available		CD10+,CD20+,CD79a+,TdT+	ALL,MDS,AML	Craniofacial/skeletal dysmorphisms

RCC = renal cell carcinoma; ALL = acute lymphoblastic leukemia; MDS = myelodysplastic syndrome; AML = acute myeloid leukemia; MCV = mean corpuscular volume. ETV6 status, hematologic phenotypes, and additional clinical features of patients in two kindreds with segregating germline *ETV6* mutations.

### Identification of germline *ETV6* mutations

DNA from 16 individuals in Kindred 1 (9 individuals with thrombocytopenia and/or ALL and 7 unaffected individuals) was subjected to Sanger sequencing for all exons of a targeted panel of leukemia-associated genes (Methods). Co-segregation of identified variants was tested using an autosomal dominant mode of inheritance. Published demographic data and medical literature were manually reviewed for all variants observed. Only one variant chr12:12,037,415 T>C satisfied the criteria of segregation as well as rarity, as evidenced by its absence in public genomic databases such as dbSNP[20], 1000 genomes[21], Exome Sequencing Project[22] and Exome Aggregation Consortium (http://exac.broadinstitute.org). This variant, identified in 9 out of 9 (100%) affected family members tested, represents a heterozygous missense *c*. *T1046C* mutation in *ETV6* (NM_001987). One individual (generation 3, individual 13) with thrombocytopenia and leukemia was not tested. This nucleotide change is predicted to result in the substitution of proline for leucine at codon 349 (L349P; Fig 1A and Table 1). Seven out of 7 (100%) unaffected family members tested exhibited a wild type(WT) *ETV6* sequence.

Fibroblast and lymphocyte DNA from the proband with ALL and parents in Kindred 2 were analyzed by clinical whole exome sequencing (Ambry Genetics, Aliso Viejo, CA, USA). The proband and his mother harbored a heterozygous deletion of 5 nucleotides (c.1153-5_1153_1delAACAG) within *ETV6*. This deletion is predicted to lead to a frameshift at codon 385 and truncation of the ETV6 protein at codon 389 (N385fs, Fig 1B and Table 1). Genome-wide DNA copy alteration analysis using single nucleotide polymorphism microarrays of the diagnostic ALL sample from the proband in Kindred 2 revealed deletion of the wild type and retention of the mutant *ETV6* allele, as well as deletions of *IKZF1*, *PAX5*, *BTG1*, and *RB1*. Other than the 2 mutations in *ETV6*, there were no pathologic genetic mutations associated with ALL or thrombocytopenia that co-segregated with disease in either kindred.

**Fig 2:**
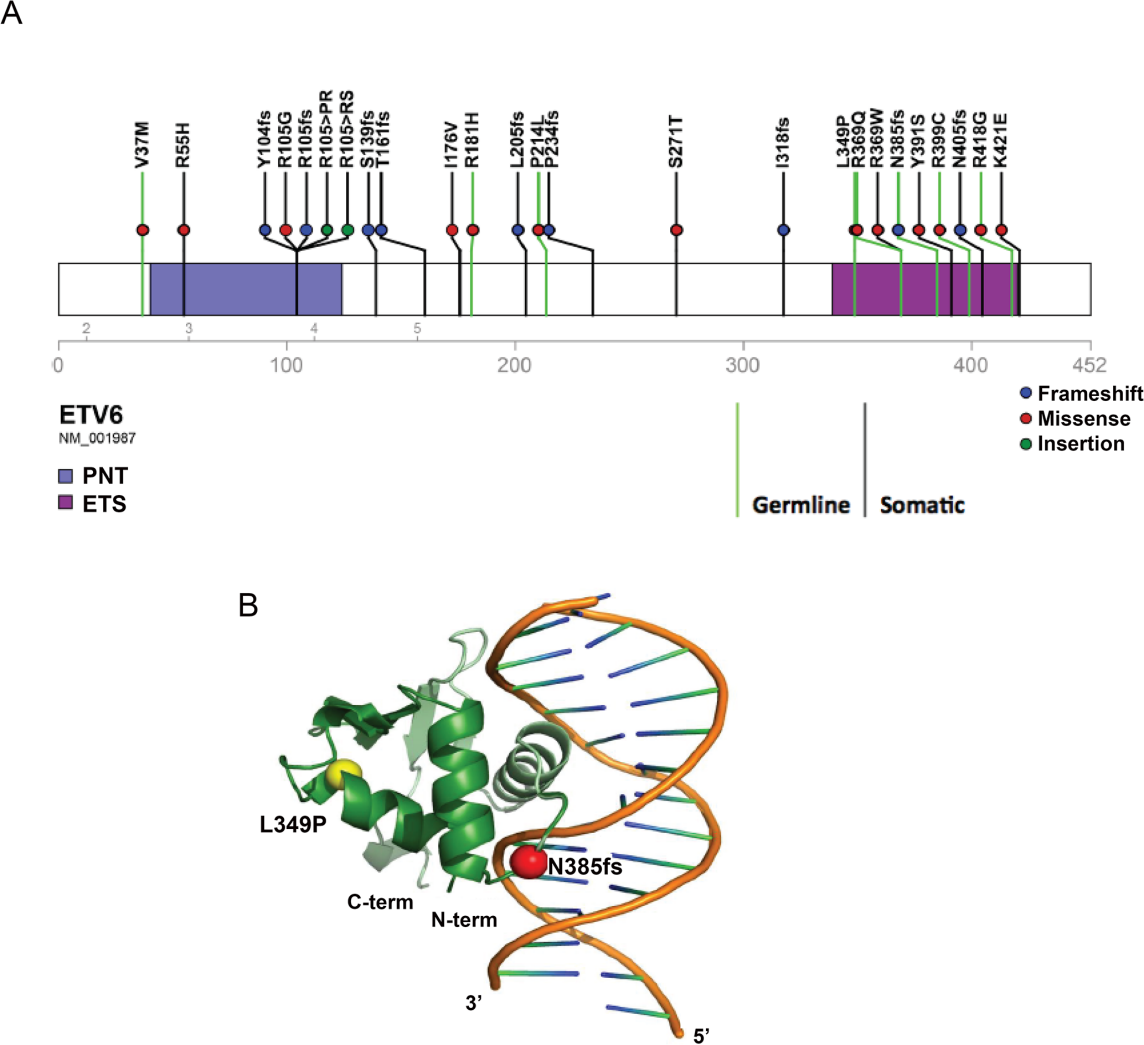
Location of somatic and germline *ETV6* mutations and structural modeling. (a) Schematic depicting the germline *ETV6* mutations detected in the MSKCC and SJCRH kindred, reported separately or somatic mutations detected as part of the Pediatric Cancer Genome Project. Somatic and germline mutations are indicated by separate green and purple lines, respectively. (b) Structural modeling of ETV6 with the germline *ETV6* L349P and N385fs mutations. The *ETV6* L349P amino acid substitution is located on an α-helix within the DNA binding domain and causes extensive kinking of the protein structure. The *ETV6* N385fs mutation results in truncation of the DNA binding domain.

Both *ETV6* variants were absent in the National Heart Lung Blood Institute (NHLBI) Exome Sequencing Project (ESP) (http://evs.gs.washington.edu/EVS/), Exome Aggregation Consortium (ExAC) (http://exac.broadinstitute.org/), or St. Jude Children’s Research Hospital–Washington University Pediatric Cancer Genome Project (PCGP) databases[23]. SIFT[24] and Polyphen prediction tools suggest the mutations to be deleterious and probably damaging to protein function. To understand how these two mutations might influence protein function, we modeled their effect on the ETV6 protein structure. Both the L349P and the N385fs mutation are located in the ETS domain of ETV6 (Fig 2A). The L349P mutation is predicted to cause significant conformational changes in areas adjacent to the ETS domain by introducing a kink in the H2 α-helix, resulting in possible ETV6 protein misfolding. The N385fs mutation affects the ETS domain and is predicted to truncate ETV6 at a region involved in DNA interaction (Fig 2B).

### Functional assessment of the *ETV6* mutations

**Fig 3:**
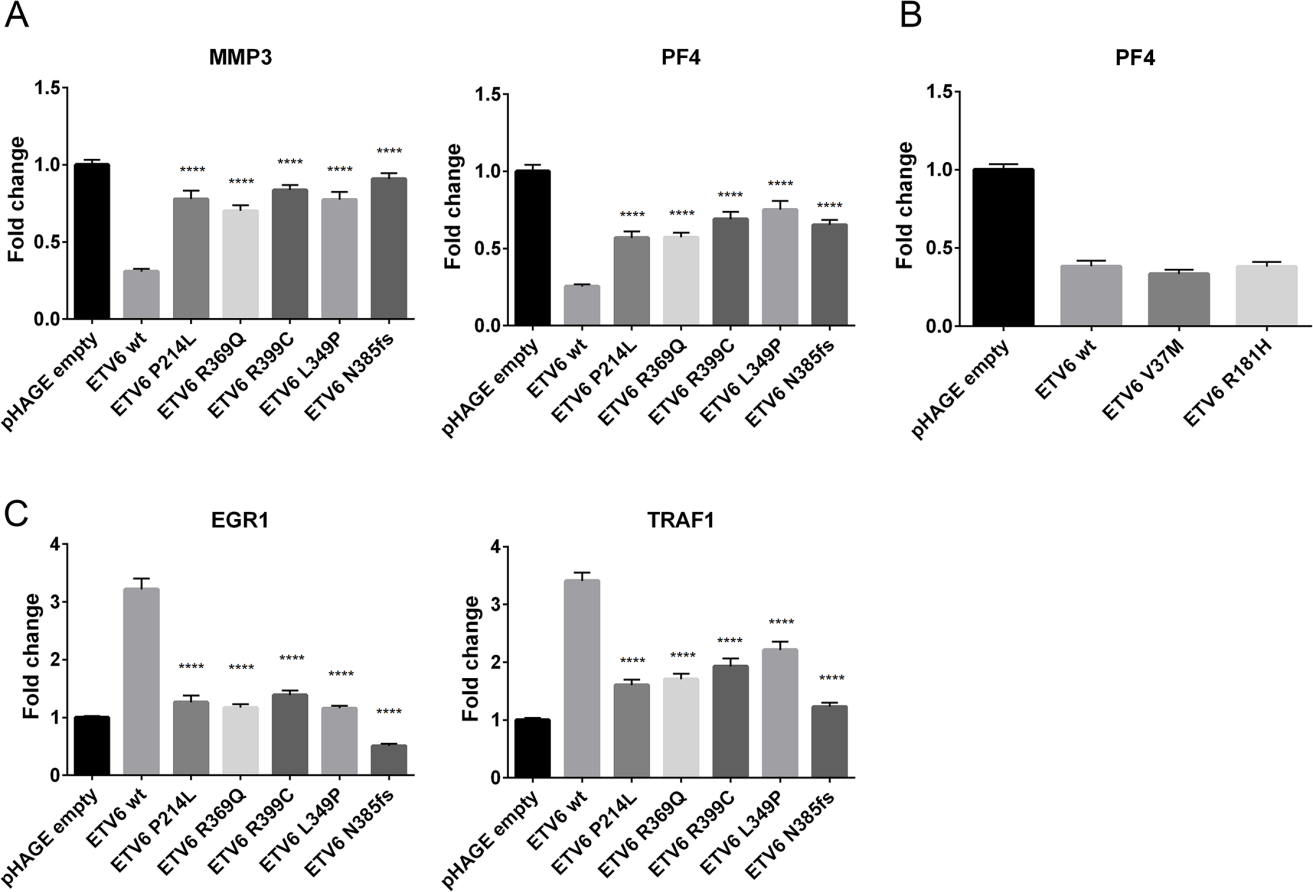
Effect of germline ETV6 mutations on transcription. (a) The effects of germline mutations on ETV6 function were examined using a Dual Luciferase Reporter Assay. Each of the mutants tested exhibited significantly (****P ≤0.0001) impaired transcriptional repression from the *PF4* and *MMP3* promoter constructs when contrasted with the WT ETV6 in the co-transfection experiment. The experiment was performed with 6 replicates for each condition and repeated 3 times. Statistical analysis was done using an unpaired t-test, the error bars show the Standard Error of Mean (SEM). (b) The effects of V37M and R181H germline mutations on ETV6 function were examined using a Dual Luciferase Reporter Assay. The experiment was performed with 6 replicates for each condition and repeated twice. Statistical analysis was done using an unpaired t-test, the error bars show the Standard Deviation (SD). (c) Quantitative PCR of ETV6 transcriptional targets *EGR1* and *TRAF1* showed reduced transcriptional abundance in the mutants when contrasted with the WT. The effect was most pronounced in the frameshift mutant. The experiment was performed in triplicate for each condition and repeated three times. Statistical analysis was done using an unpaired t-test, the error bars show the Standard Error of Mean (SEM).

To evaluate the functional consequences of these mutations, we first assessed whether L349P and N385fs might impair transcriptional repression by ETV6. HeLa cells were transiently co-transfected with constructs encoding the WT or mutant *ETV6*, as well as constructs containing the *PF4* or *MMP3* promoters, which harbor multiple ETS binding sites and are natural ETV6 targets. We compared the results to those obtained using other recently described germline *ETV6* variants, P214L, R369Q, R399C [17]. As expected, WT ETV6 repressed expression of both reporters (Fig 3A), while each of the *ETV6* mutants exhibited significantly decreased repression. To further explore the effects of the *ETV6* mutations, we analyzed the expression of *EGR1* and *TRAF1*, genes that are normally upregulated by WT ETV6 [17]. Consistent with published reports, *EGR1* and *TRAF1* were upregulated 3-fold in cells transfected with WT *ETV6*. In contrast, the mutants induced minimal to no upregulation for both of these target genes (Fig 3C). Indeed, the levels were significantly reduced compared to WT ETV6. In each of these assays, we observed comparable levels of WT and ETV6 mutant mRNA transcripts (Supporting Information 1). Thus, transcript stability appears to be unaffected by the *ETV6* mutations.

**Fig 4:**
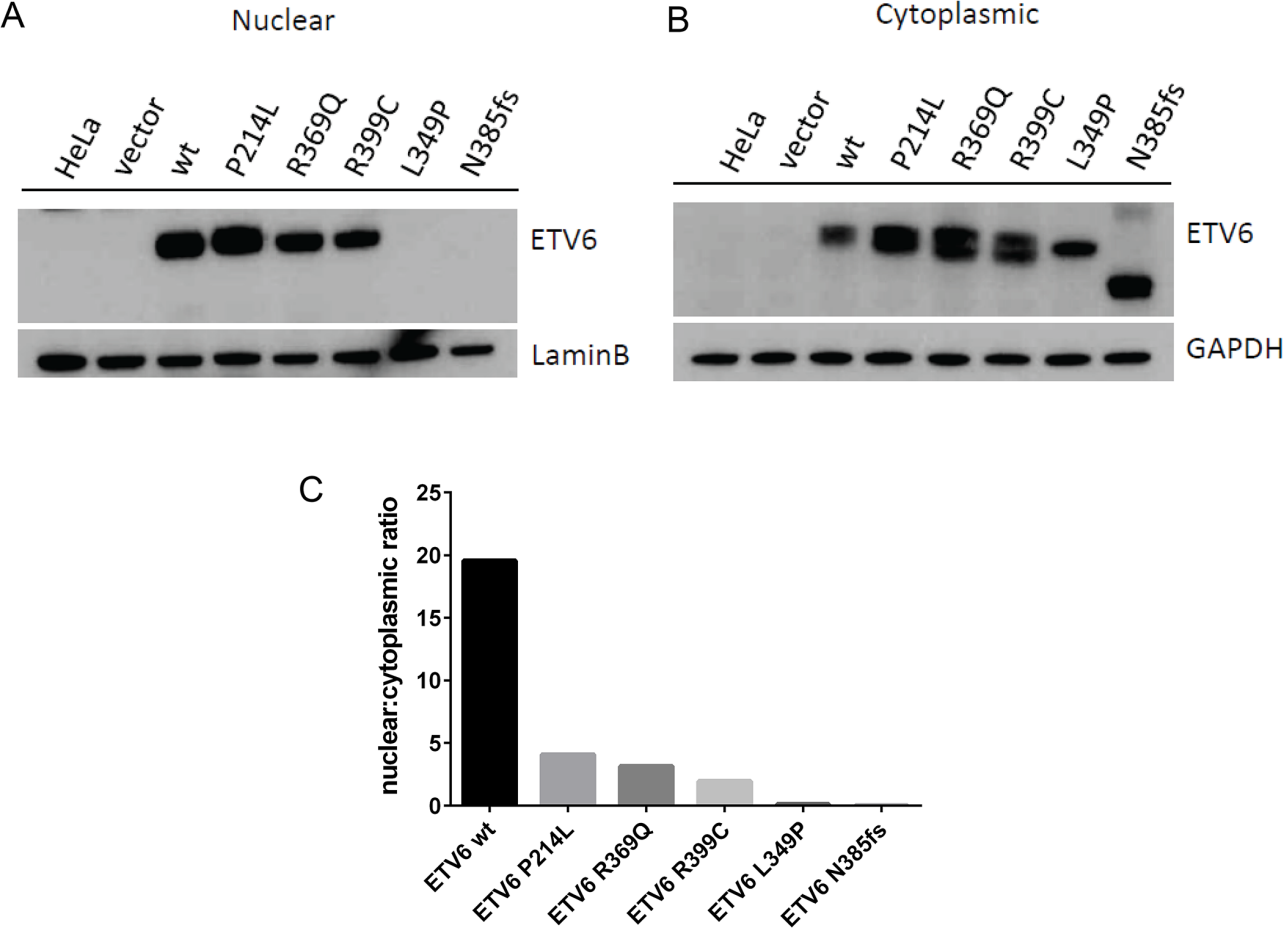
Germline *ETV6* mutations impair localization of the ETV6 protein. Western blots of HeLa cell fractions probed for ETV6 protein show (a) presence of ETV6 within the cells transiently overexpressing ETV6 WT and the P214L, R369Q and R339C mutants within the nucleus. Both the L349P and N385fs mutant were not detected in the nuclear fraction. (b) Presence of ETV6 protein is abundant in the cytoplasmic fraction. The frameshift mutant N385fs showed a protein product that was smaller (45kDa) than the full-length protein (53kDa). (c) Densitometric analysis of the western blots shows that the WT localization is predominantly nuclear, while the L349P and N385fs are cytoplasmic. Other mutants P214L, R369Q and R339C show localization to a lesser extent in the nucleus.

To examine whether the L349P and N385fs mutations negatively impact translation or alter subcellular localization of the ETV6 protein, we performed cell fractionation assays and western blotting of HeLa cells transiently transfected to express WT or mutant ETV6. Both proteins were detectable by Western blotting, with a smaller product observed for the N385fs mutation. Both mutants were undetectable in the nucleus (Fig 4A), but detected within the cytoplasmic fraction (Fig 4B), This is in contrast to the described mutants P214L, R369Q and R399C, which were detected in cytoplasmic as well as nuclear fractions. These patterns were quantitated and confirmed by measuring the nuclear to cytoplasmic ratio (Fig 4C).

### Evaluation of the incidence of germline *ETV6* mutations

Fusions involving *ETV6* in leukemia have long been recognized [25–27]. Other mutation types, including single nucleotide variations, insertions, deletions, frame-shifts and non-sense alterations are also becoming increasingly evident in hematologic malignancies[17,18,28]. We performed additional sequence analysis on exons 5–8 of *ETV6* in unrelated probands from 27 unrelated kindreds with a family history of ALL, but identified no mutations in this region of *ETV6*. To further characterize the spectrum of germline and somatic *ETV6* mutations that contribute to childhood leukemia, we screened a cohort of 588 leukemia patients evaluated through the PCGP, a genomic sequencing effort involving pediatric cancers [4,28–42](accession# EGAS00001000348, EGAS00001000654, EGAS00001000380, EGAS00001000253, EGAS00001000246, EGAS00001000447). Seventeen distinct somatic *ETV6* variants and two rare germline variants were identified (V37M, R181H; Fig 2A). Both rare variants occurred in patients with B-ALL, but with no evidence for loss or mutation of the WT *ETV6* allele within the leukemia samples. In one of these cases, there was a secondary vulvar squamous cell carcinoma. There was nofamily history of leukemia or thrombocytopenia in either of these cases. Luciferase assays performed on these variants showed no significant changes in transcriptional repression activity when compared to WT *ETV6* (Fig 3B). We queried several public variant databases for the presence of these two variants. The 1000 genomes project has a total of 2,819 samples from the world’s major populations. The current version of the Exome sequencing project (EVS/ESP) has a set of 2,203 African-American and 4300 European-American unrelated individuals, totaling 6,503 samples. The Exome aggregation consortium (ExAC) has 60,706 unrelated individuals sequenced as part of various disease-specific and population genetic studies. In total, we have queried over 140,000 chromosomes. The V37M variant (chr12:11905459G>A) was seen only in the ExAC data at an allele frequency of 1.649x10^-05^ and the R181H variant (chr12:12022436 G>A) was found at an allele frequency of 1.071x10^-04^ in ExAC. It was also found 2 times in NHLBI-ESP (AF = 1.162x10^-04^) and assigned as rs150089916. While V37M was predicted *in silico* as benign by SIFT, R181H was classified as deleterious. Based on these preliminary findings the clinical significance of these two additional rare germline variants remains to be determined and at this time is classified as variants of unknown significance.

## Discussion

*ETV6* encodes an ETS family transcription factor that is frequently rearranged or fused with other genes in human leukemias of myeloid or lymphoid origin[28]. Also known as the *TEL* oncogene, *ETV6* is a sequence specific transcriptional repressor, regulated by auto-inhibition and self-association[43,44]. Descriptions of *ETV6* largely focus on the *ETV6/RUNX1* fusion, which is a product of a t(12;21) chromosomal translocation, the most common genetic abnormality in pediatric ALL[25]. While somatic deletions or mutations in *ETV6* are increasingly recognized in ALL, nothing is known regarding the impact of germline *ETV6* mutations[17,28]. Here we extend the description of the clinical phenotype and functional effects associated with novel germline *ETV6* L349P and *ETV6* N385fs mutations, both of which reside in the highly conserved ETS DNA binding domain and co-segregate with disease in 2 unrelated kindreds affected by thrombocytopenia and ALL.

In both kindreds *ETV6* mutations were inherited in an autosomal dominant manner with variable expression of thrombocytopenia and/or ALL. There was no evidence for parent of origin or sex-delimited expression, as males and females equally transmitted the putative predisposing alleles with associated phenotypes manifesting in daughters as well as sons. Interestingly, in addition to his leukemia, the proband in Kindred 2 exhibited craniofacial and musculoskeletal anomalies (anterior placement of the right ear, downward shaped mouth, joint hypermobility and CNS heterotopias seen on magnetic resonance imaging). No other obvious pathogenic variants were identified in this individual by whole exome sequencing. In addition to atypical physical features, the proband in Kindred 2 developed grade 3 myelosuppression following exposure to anti-metabolite therapy; this feature of chemotherapy hypersensitivity was shared by another patient with T-/myeloid mixed phenotype leukemia and a germline *ETV6* mutation (P214L) [17]. In addition, two of the three individuals affected with ALL and harboring *ETV6* mutations in the kindreds reported here required bone marrow transplantation, and 1 of the 3 expired from disease, in contrast to the 90% rate of cure with chemotherapy alone in more typical ALL. Whether germline *ETV6* mutations might serve as markers for toxicity and outcome will require larger studies controlling for other prognostic variables.

*In vitro* studies revealed impaired function of the ETV6 mutants identified in both kindreds. While *ETV6* L349P and N385fs exhibited normal mRNA levels, both mutations were associated with decreased transcriptional regulation (repression and activation). Structural modeling suggests that both *ETV6* mutations would impair transcriptional activity by altering the conformation of the ETV6 protein or truncating it within the DNA binding domain. Interestingly, neither mutant localized to the nucleus. Although the precise mechanism for this behavior remains unclear, it seems likely that these two mutations may affect intracellular transport. Consistent with its putative role as a tumor suppressor, examination of the diagnostic leukemia sample in the proband from Kindred 2 revealed retention of the mutant and deletion of the WT *ETV6* allele. Our findings are in agreement with 2 recent reports describing additional *ETV6* mutations, including R399C, R369Q[17] and R418G[18] in the ETS DNA binding domain and P214L[17,18], located in a serine-proline phosphorylation motif present in the internal linker domain. In the 3 reports of germline *ETV6* mutations to date (including the current series), a mixed phenotype of thrombocytopenia and ALL is observed. An association with elevated MCV was not observed in 3 cases included here, which is in contrast to one of the other recent reports[18]. The 2 additional germline variants reported here in patients with ALL (V37M and R181H) did not impair transcriptional repression of ETV6. While this was expected given that these mutations are not located in or close by the ETS DNA binding domain, we cannot exclude that these variants impair ETV6 function on another functional level.

The discovery of mutations in *ANKRD26*, *RUNX1*, and the ETS family transcription factors has led to an increased understanding of the genetic basis of hereditary syndromes involving thrombocytopenia, red cell macrocytosis and leukemia [9,10,17,18] and of the pathways regulated by these genes [17,45]. Constitutional alterations in *RUNX1* predispose individuals to thrombocytopenia and hematological malignancies, mainly myelodysplastic syndrome and AML, but also T-ALL [3,9,10,46,47]. Mutations in *RUNX1* have been shown to result in either haploinsufficiency or can act in a dominant-negative manner, the latter resulting in an increased risk of hematological malignancies [48,49]. Inherited mutations in *ANKRD26* [10,45], which is transcriptionally regulated by *RUNX1* lead to a similar clinical phenotype, in which thrombocytopenia is often associated with AML and in some cases, with chronic myelogenous leukemia, chronic lymphocytic leukemia and myelodysplastic syndrome [38]. However, there remain additional kindreds affected by thrombocytopenia and/or leukemia that do not demonstrate germline mutations of *RUNX1* or *ANKRD26*. Our data suggest that at least a proportion of these cases result from *ETV6* mutations. To date, it is not known whether the *ETV6* pathway contributes to non-leukemic cancer phenotypes. We observed no pathogenic germline *ETV6* mutations in children with cancers other than ALL in the PCGP. Therefore, the contribution of *ETV6* mutations to solid tumor predisposition remains to be determined.

Improved understanding of the heritable nature of childhood cancers has important clinical implications pertaining to genetic counseling and testing of other family members, therapeutic decisions, donor selection for hematopoietic transplantation, and long-term monitoring for therapy-associated or second primary neoplasms [17,50,51]. Evaluation for germline alterations of *ETV6* is therefore warranted in families with acute lymphoblastic leukemia, particularly when there is preceding evidence of thrombocytopenia.

## Materials & Methods

### Patients and controls

All individuals analyzed for purposes of our research were formally consented to Memorial Sloan Kettering Cancer Center’s IRB approved research Protocol, Protocol #00–069, “*Ascertainment of Families for Genetic Studies of Familial Lymphoproliferative Disorders”*, or St. Jude Children’s Research Hospital IRB approved research Protocols NR14-132, “*Case report of child with novel ETV6 mutation associated with development of leukemia”* and/or NR14-162, “*ETV6 germline variants in children with acute lymphoblastic leukemia”*, respectively. For Kindred 1, we included 9 affected and 7 unaffected individuals for sequencing. For Kindred 2, we included the proband with ALL, his mother with thrombocytopenia and his unaffected father. For both kindreds, the presence and subtype of leukemia were confirmed by review of pathology reports, while thrombocytopenia was confirmed by medical history.

#### Sequencing

DNA from the proband of Kindred 1 was collected by buccal swab and extracted using the buccal swab DNA isolation kit, (Isohelix, Cat-# DDK-50SK2). DNA from all other family members in Kindred 1 was extracted from saliva using the Oragene DNA extraction kit (DNA Genotek, Cat# OG-250). DNA sequencing of all exons of the leukemia associated genes *PAX5*, *ETV6*, *HOXA11*, *CDKN2A*, *TAL1* and *ERG* was performed using Sanger sequencing. Clinical exome sequencing (Kindred 2) was performed by Ambry Genetics (Ambry Genetics, Aliso Viejo, CA, USA). To this end, DNA libraries were prepared using 2μg of blood derived DNA (Paired End DNA Sample preparation Kit; Illumina). DNA was fragmented and libraries prepared. Target enrichment was carried out utilizing the TruSeq Exome enrichment Kit, which targets 62 megabases of the human genome. Captured DNA libraries were PCR amplified using the supplied paired end PCR primers.

#### Variant assessment

For Kindred 2, sequence reads were aligned to the human genome reference GRCh37.1 using the Burrows-Wheeler Aligner (BWA) [52], The resulting binary alignment format (BAM files) were jointly called for single nucleotide variants and insertion/deletions (indels) using the Genome Analysis Toolkit (GATK) v.3.1 [53], while structural variations were detected using Clipping Reveals Structure (CREST) [37,54]. Variant level annotations were performed using *in-silico* tools, such as ANNOVAR[55]. These annotations were used to predict the effects of identified germline variants on gene function and the relevant medical literature was reviewed. Variants were manually reviewed against the medical literature and disease locus specific databases.

#### Plasmids

The pHAGE-CMV-MCS-IRES-ZsGreen lentiviral expression plasmids containing WT human *ETV6* and the *ETV6* P214L, R369Q and R399C mutants as well as the pGL3-MMP3, pGL3-PF4 and pCS2-Renilla luciferase plasmids were provided by A. Shimamura[17]. *ETV6* L349P and N385fs mutants were generated from the WT *ETV6* plasmid using QuickChange II XL Site-Directed Mutagenesis Kit (Agilent).

#### Cell culture and transfections

The HeLa cells used in this study (a gift from A.Ventura, MSKCC) were derived from a subculture of the HeLa cell line (ATCC Cat# CCL-2) and subsequently tested for mycoplasma before being used in experiments. These cells were cultured in Dulbecco’s Modified Eagle Medium supplemented with 10%FBS, 1mM L-Glutamine and 1% penicillin-streptomycin. Cell cultures were maintained in a humidified incubator at 37°C in 5% CO_2._ Transfections were carried out with FuGENE 6 transfection reagent (Promega) or Lipofectamine 2000 (Life Technologies) according to the manufacturer’s instructions.

#### Real-time PCR

RNA was extracted 24 h after transfection using the RNeasy Mini Kit (Qiagen) and reverse transcribed with the ReadyScript cDNA Synthesis Mix (Sigma-Aldrich). Quantitative Real-time PCR analyses were performed on an ABI PRISM 7900HT Sequence Detection System using the *Power* SYBR Green PCR Master Mix (Life Technologies) according to the manufacturer’s instructions. Following initial incubation for 10 min at 95°C, amplification was performed for 40 cycles at 95°C for 15 s and 60°C for 1 min. The Rpl32 gene was used as the internal standard and normalization for transfection efficiency was carried out with ZsGreen. Analysis was performed as per the based on the comparative C_T_ method. The following primer sequences were used:

Rpl32 F, 5’-CATCTCCTTCTCGGCATCA-3’;

Rpl32 R, 5’-AACCCTGTTGTCAATGCCTC-3’;

ZsGreen F, 5’-CTACTTCAAGAACTCCTGCCC-3’;

ZsGreen R, 5’-TCGTGGTACATGCAGTTCTC-3’;

TRAF1 qRT F, 5’-AAGATCACCAATGTCACCAGG-3’;

TRAF1 qRT R, 5’-GCCATCTCCATTCAGGTACAG-3’;

EGR1 qRT F, 5’-CAGCACCTTCAACCCTCAG-3’;

EGR1 qRT R, 5’-AGTCGAGTGGTTTGGCTG-3’;

#### Western blotting

To isolate protein lysates from nuclear and cytoplasmic subcellular fractions, transfected HeLa cells were lysed 48 hours after transfection (transfection efficiency was measured by ZsGreen positive cells using Guava easyCyte Flow Cytometer) and fractionation was performed using the NE-PER Nuclear and Cytoplasmic Extraction Reagents (Pierce). Samples were run on 4–12% gradient Bis-Tris SDS-PAGE gels, transferred onto PVDF membranes (Bio-Rad) and probed with antibodies against ETV6 (AF7945; 1:200; R&D systems), GAPDH (V-18; 1:200) and Lamin B (C-20; 1:400) (Santa Cruz Biotechnology). Probes were detected using ECL Prime Western Blotting Detection Reagent (GE Healthcare).

#### Luciferase assay

HeLa cells were co-transfected with pHAGE expression constructs, pGL3 reporter constructs and pCS2-Renilla luciferase construct and were harvested 48h after transfection using passive lysis buffer (Promega). Measurement of Firefly and Renilla luciferase expression levels was performed using the Dual-Luciferase Reporter Assay System (Promega) on a GloMax-96 Microplate Luminometer (Promega).

## Supporting Information

S1 Fig Quantitative PCR analysis. Quantitative PCR analysis using cDNA derived from transiently transfected HeLa cells reveals comparable transcript levels for WT ETV6, the L349P and N385fs mutants identified in the MSKCC and SJCRH kindreds, as well as mutants described in a recent separate report (P214L, R369Q, R399C) ^17^.
(TIF)

## References

[1] Malkin D, Nichols KE, Zelley K, Schiffman JD (2014) Predisposition to pediatric and hematologic cancers: a moving target. Am Soc Clin Oncol Educ Book: e44-55. 10.14694/EdBook_AM.2014.34.e44 24857136

[2] Pui C-H (2012) Childhood leukemias Cambridge, UK; New York: Cambridge University Press xi, 880 p., 824 p. of col. plates p.

[3] Arepally G, Rebbeck TR, Song W, Gilliland G, Maris JM, et al (1998) Evidence for genetic homogeneity in a familial platelet disorder with predisposition to acute myelogenous leukemia (FPD/AML). Blood 92: 2600–2602. 9746808

[4] Holmfeldt L, Wei L, Diaz-Flores E, Walsh M, Zhang J, et al (2013) The genomic landscape of hypodiploid acute lymphoblastic leukemia. Nat Genet 45: 242–252. 10.1038/ng.2532 23334668 PMC3919793

[5] Shah S, Schrader KA, Waanders E, Timms AE, Vijai J, et al (2013) A recurrent germline PAX5 mutation confers susceptibility to pre-B cell acute lymphoblastic leukemia. Nat Genet 45: 1226–1231. 10.1038/ng.2754 24013638 PMC3919799

[6] Smith ML, Cavenagh JD, Lister TA, Fitzgibbon J (2004) Mutation of CEBPA in familial acute myeloid leukemia. N Engl J Med 351: 2403–2407. 15575056 10.1056/NEJMoa041331

[7] Stieglitz E, Loh ML (2013) Genetic predispositions to childhood leukemia. Ther Adv Hematol 4: 270–290. 10.1177/2040620713498161 23926459 PMC3734905

[8] Powell BC, Jiang L, Muzny DM, Trevino LR, Dreyer ZE, et al (2013) Identification of TP53 as an acute lymphocytic leukemia susceptibility gene through exome sequencing. Pediatr Blood Cancer 60: E1–3. 10.1002/pbc.24417 23255406 PMC3926299

[9] Song WJ, Sullivan MG, Legare RD, Hutchings S, Tan X, et al (1999) Haploinsufficiency of CBFA2 causes familial thrombocytopenia with propensity to develop acute myelogenous leukaemia. Nat Genet 23: 166–175. 10508512 10.1038/13793

[10] Noris P, Perrotta S, Seri M, Pecci A, Gnan C, et al (2011) Mutations in ANKRD26 are responsible for a frequent form of inherited thrombocytopenia: analysis of 78 patients from 21 families. Blood 117: 6673–6680. 10.1182/blood-2011-02-336537 21467542

[11] Ciovacco WA, Raskind WH, Kacena MA (2008) Human phenotypes associated with GATA-1 mutations. Gene 427: 1–6. 10.1016/j.gene.2008.09.018 18930124 PMC2601579

[12] Ballmaier M, Schulze H, Strauss G, Cherkaoui K, Wittner N, et al (1997) Thrombopoietin in patients with congenital thrombocytopenia and absent radii: elevated serum levels, normal receptor expression, but defective reactivity to thrombopoietin. Blood 90: 612–619. 9226161

[13] Go RS, Johnston KL (2003) Acute myelogenous leukemia in an adult with thrombocytopenia with absent radii syndrome. Eur J Haematol 70: 246–248. 12656750 10.1034/j.1600-0609.2003.00054.x

[14] Fujino T, Suzuki A, Ito Y, Ohyashiki K, Hatano Y, et al (2002) Single-translocation and double-chimeric transcripts: detection of NUP98-HOXA9 in myeloid leukemias with HOXA11 or HOXA13 breaks of the chromosomal translocation t(7;11)(p15;p15). Blood 99: 1428–1433. 11830496 10.1182/blood.v99.4.1428

[15] Albers CA, Newbury-Ecob R, Ouwehand WH, Ghevaert C (2013) New insights into the genetic basis of TAR (thrombocytopenia-absent radii) syndrome. Curr Opin Genet Dev 23: 316–323. 10.1016/j.gde.2013.02.015 23602329

[16] Balduini CL, Savoia A (2012) Genetics of familial forms of thrombocytopenia. Hum Genet 131: 1821–1832. 10.1007/s00439-012-1215-x 22886561

[17] Zhang MY, Churpek JE, Keel SB, Walsh T, Lee MK, et al (2015) Germline ETV6 mutations in familial thrombocytopenia and hematologic malignancy. Nat Genet 47: 180–185. 10.1038/ng.3177 25581430 PMC4540357

[18] Noetzli L, Lo RW, Lee-Sherick AB, Callaghan M, Noris P, et al (2015) Germline mutations in ETV6 are associated with thrombocytopenia, red cell macrocytosis and predisposition to lymphoblastic leukemia. Nat Genet. 10.1038/ng.3253 PMC4631613 25807284

[19] Walsh T, Casadei S, Lee MK, Pennil CC, Nord AS, et al (2011) Mutations in 12 genes for inherited ovarian, fallopian tube, and peritoneal carcinoma identified by massively parallel sequencing. Proc Natl Acad Sci U S A 108: 18032–18037. 10.1073/pnas.1115052108 22006311 PMC3207658

[20] Sherry ST, Ward MH, Kholodov M, Baker J, Phan L, et al (2001) dbSNP: the NCBI database of genetic variation. Nucleic Acids Res 29: 308–311. 11125122 10.1093/nar/29.1.308 PMC29783

[21] Genomes Project C, Abecasis GR, Auton A, Brooks LD, DePristo MA, et al (2012) An integrated map of genetic variation from 1,092 human genomes. Nature 491: 56–65. 10.1038/nature11632 23128226 PMC3498066

[22] Exome Variant Server, NHLBI GO Exome Sequencing Project (ESP), Seattle, WA (URL: http://evs.gs.washington.edu/EVS/).

[23] Downing JR, Wilson RK, Zhang J, Mardis ER, Pui CH, et al (2012) The Pediatric Cancer Genome Project. Nat Genet 44: 619–622. 10.1038/ng.2287 22641210 PMC3619412

[24] P. C. Ng SH (2003) SIFT: Predicting amino acid changes that affect protein function. Nucleic Acides Res 31: 3812–3814. 12824425 10.1093/nar/gkg509 PMC168916

[25] Shurtleff SA, Buijs A, Behm FG, Rubnitz JE, Raimondi SC, et al (1995) TEL/AML1 fusion resulting from a cryptic t(12;21) is the most common genetic lesion in pediatric ALL and defines a subgroup of patients with an excellent prognosis. Leukemia 9: 1985–1989. 8609706

[26] Barbany G, Andersen MK, Autio K, Borgstrom G, Franco LC, et al (2012) Additional aberrations of the ETV6 and RUNX1 genes have no prognostic impact in 229 t(12;21)(p13;q22)-positive B-cell precursor acute lymphoblastic leukaemias treated according to the NOPHO-ALL-2000 protocol. Leuk Res 36: 936–938. 10.1016/j.leukres.2012.03.024 22521551

[27] De Braekeleer E, Douet-Guilbert N, Morel F, Le Bris MJ, Basinko A, et al (2012) ETV6 fusion genes in hematological malignancies: a review. Leuk Res 36: 945–961. 10.1016/j.leukres.2012.04.010 22578774

[28] Wang Q, Dong S, Yao H, Wen L, Qiu H, et al (2014) ETV6 mutation in a cohort of 970 patients with hematologic malignancies. Haematologica 99: e176–178. 10.3324/haematol.2014.104406 24997145 PMC4181263

[29] Parker M, Chen X, Bahrami A, Dalton J, Rusch M, et al (2012) Assessing telomeric DNA content in pediatric cancers using whole-genome sequencing data. Genome Biol 13: R113 10.1186/gb-2012-13-12-r113 23232254 PMC3580411

[30] Wu G, Diaz AK, Paugh BS, Rankin SL, Ju B, et al (2014) The genomic landscape of diffuse intrinsic pontine glioma and pediatric non-brainstem high-grade glioma. Nat Genet 46: 444–450. 10.1038/ng.2938 24705251 PMC4056452

[31] Zhang J, Ding L, Holmfeldt L, Wu G, Heatley SL, et al (2012) The genetic basis of early T-cell precursor acute lymphoblastic leukaemia. Nature 481: 157–163. 10.1038/nature10725 22237106 PMC3267575

[32] Cancer Genome Atlas Research N (2013) Genomic and epigenomic landscapes of adult de novo acute myeloid leukemia. N Engl J Med 368: 2059–2074. 10.1056/NEJMoa1301689 23634996 PMC3767041

[33] Lu C, Zhang J, Nagahawatte P, Easton J, Lee S, et al. (2014) The Genomic Landscape of Childhood and Adolescent Melanoma. J Invest Dermatol. 10.1038/jid.2014.425 PMC4340976 25268584

[34] Chen X, Bahrami A, Pappo A, Easton J, Dalton J, et al (2014) Recurrent somatic structural variations contribute to tumorigenesis in pediatric osteosarcoma. Cell Rep 7: 104–112. 10.1016/j.celrep.2014.03.003 24703847 PMC4096827

[35] Gruber TA, Larson Gedman A, Zhang J, Koss CS, Marada S, et al (2012) An Inv(16)(p13.3q24.3)-encoded CBFA2T3-GLIS2 fusion protein defines an aggressive subtype of pediatric acute megakaryoblastic leukemia. Cancer Cell 22: 683–697. 10.1016/j.ccr.2012.10.007 23153540 PMC3547667

[36] Zhang J, Mullighan CG, Harvey RC, Wu G, Chen X, et al (2011) Key pathways are frequently mutated in high-risk childhood acute lymphoblastic leukemia: a report from the Children's Oncology Group. Blood 118: 3080–3087. 10.1182/blood-2011-03-341412 21680795 PMC3175785

[37] Huether R, Dong L, Chen X, Wu G, Parker M, et al (2014) The landscape of somatic mutations in epigenetic regulators across 1,000 paediatric cancer genomes. Nat Commun 5: 3630 10.1038/ncomms4630 24710217 PMC4119022

[38] Robinson G, Parker M, Kranenburg TA, Lu C, Chen X, et al (2012) Novel mutations target distinct subgroups of medulloblastoma. Nature 488: 43–48. 10.1038/nature11213 22722829 PMC3412905

[39] Wu G, Broniscer A, McEachron TA, Lu C, Paugh BS, et al (2012) Somatic histone H3 alterations in pediatric diffuse intrinsic pontine gliomas and non-brainstem glioblastomas. Nat Genet 44: 251–253. 10.1038/ng.1102 22286216 PMC3288377

[40] Zhang J, Benavente CA, McEvoy J, Flores-Otero J, Ding L, et al (2012) A novel retinoblastoma therapy from genomic and epigenetic analyses. Nature 481: 329–334. 10.1038/nature10733 22237022 PMC3289956

[41] Roberts KG, Li Y, Payne-Turner D, Harvey RC, Yang YL, et al (2014) Targetable kinase-activating lesions in Ph-like acute lymphoblastic leukemia. N Engl J Med 371: 1005–1015. 10.1056/NEJMoa1403088 25207766 PMC4191900

[42] Chen X, Stewart E, Shelat AA, Qu C, Bahrami A, et al (2013) Targeting oxidative stress in embryonal rhabdomyosarcoma. Cancer Cell 24: 710–724. 10.1016/j.ccr.2013.11.002 24332040 PMC3904731

[43] Green SM, Coyne HJ 3rd, McIntosh LP, Graves BJ (2010) DNA binding by the ETS protein TEL (ETV6) is regulated by autoinhibition and self-association. J Biol Chem 285: 18496–18504. 10.1074/jbc.M109.096958 20400516 PMC2881775

[44] Wang LC, Swat W, Fujiwara Y, Davidson L, Visvader J, et al (1998) The TEL/ETV6 gene is required specifically for hematopoiesis in the bone marrow. Genes Dev 12: 2392–2402. 9694803 10.1101/gad.12.15.2392 PMC317042

[45] Bluteau D, Balduini A, Balayn N, Currao M, Nurden P, et al (2014) Thrombocytopenia-associated mutations in the ANKRD26 regulatory region induce MAPK hyperactivation. J Clin Invest 124: 580–591. 10.1172/JCI71861 24430186 PMC3904625

[46] Mullighan CG, Zhang J, Kasper LH, Lerach S, Payne-Turner D, et al (2011) CREBBP mutations in relapsed acute lymphoblastic leukaemia. Nature 471: 235–239. 10.1038/nature09727 21390130 PMC3076610

[47] Liew E, Owen C (2011) Familial myelodysplastic syndromes: a review of the literature. Haematologica 96: 1536–1542. 10.3324/haematol.2011.043422 21606161 PMC3186316

[48] Cohen MM Jr. (2009) Perspectives on RUNX genes: an update. Am J Med Genet A 149A: 2629–2646. 10.1002/ajmg.a.33021 19830829

[49] Matheny CJ, Speck ME, Cushing PR, Zhou Y, Corpora T, et al (2007) Disease mutations in RUNX1 and RUNX2 create nonfunctional, dominant-negative, or hypomorphic alleles. EMBO J 26: 1163–1175. 17290219 10.1038/sj.emboj.7601568 PMC1852839

[50] Offit K, Sagi M, Hurley K (2006) Preimplantation genetic diagnosis for cancer syndromes: a new challenge for preventive medicine. JAMA 296: 2727–2730. 17164459 10.1001/jama.296.22.2727

[51] Savage SA, Alter BP (2009) Dyskeratosis Congenital. Hematology-Oncology Clinics of North America 23: 215–+. 10.1016/j.hoc.2009.01.003 19327580 PMC2702847

[52] Li H, Durbin R (2010) Fast and accurate long-read alignment with Burrows-Wheeler transform. Bioinformatics 26: 589–595. 10.1093/bioinformatics/btp698 20080505 PMC2828108

[53] DePristo MA, Banks E, Poplin R, Garimella KV, Maguire JR, et al (2011) A framework for variation discovery and genotyping using next-generation DNA sequencing data. Nat Genet 43: 491–498. 10.1038/ng.806 21478889 PMC3083463

[54] Wang J, Mullighan CG, Easton J, Roberts S, Heatley SL, et al (2011) CREST maps somatic structural variation in cancer genomes with base-pair resolution. Nat Methods 8: 652–654. 10.1038/nmeth.1628 21666668 PMC3527068

[55] Wang K, Li M, Hakonarson H (2010) ANNOVAR: functional annotation of genetic variants from high-throughput sequencing data. Nucleic Acids Res 38: e164 10.1093/nar/gkq603 20601685 PMC2938201

