# Supplementary figures and images for: Germline ETV6 Mutations Confer Susceptibility to Acute Lymphoblastic Leukemia and Thrombocytopenia
Source: PLoS Genet. 2015 Jun 23;11(6):e1005262. doi: 10.1371/journal.pgen.1005262 (PMC4477877; doi:10.1371/journal.pgen.1005262)

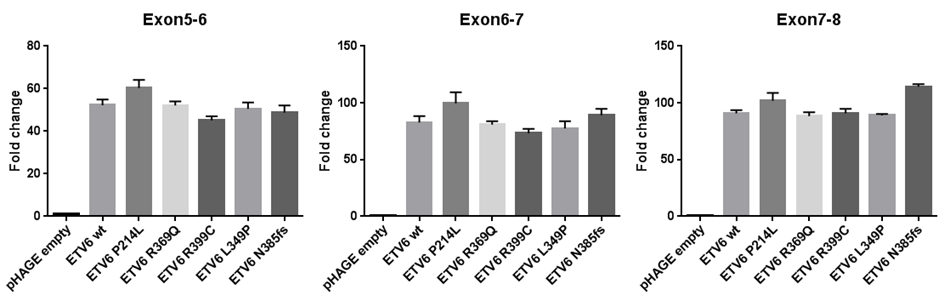

Supplement: S1 Fig — Quantitative PCR analysis using cDNA derived from transiently transfected HeLa cells reveals comparable transcript levels for WT ETV6, the L349P and N385fs mutants identified in the MSKCC and SJCRH kindreds, as well as mutants described in a recent separate report (P214L, R369Q, R399C) 17. (TIF) [file pgen.1005262.s001.tif]
